# Use of Oral Cholera Vaccine and Knowledge, Attitudes, and Practices Regarding Safe Water, Sanitation and Hygiene in a Long-Standing Refugee Camp, Thailand, 2012-2014

**DOI:** 10.1371/journal.pntd.0005210

**Published:** 2016-12-19

**Authors:** Heather M. Scobie, Christina R. Phares, Kathleen A. Wannemuehler, Edith Nyangoma, Eboni M. Taylor, Anna Fulton, Nuttapong Wongjindanon, Naw Rody Aung, Phillipe Travers, Kashmira Date

**Affiliations:** 1 Global Immunization Division, Centers for Disease Control and Prevention, Atlanta, Georgia, United States of America; 2 Epidemic Intelligence Service, Centers for Disease Control and Prevention, Atlanta, Georgia, United States of America; 3 Thailand Ministry of Public Health – U.S. Centers for Disease Control and Prevention Collaboration, Nonthaburi, Thailand; 4 Division of Global Migration and Quarantine, Centers for Disease Control and Prevention, Atlanta, Georgia, United States of America; 5 Première Urgence-Aide Médicale Internationale, Mae Sot, Thailand; University of California San Diego School of Medicine, UNITED STATES

## Abstract

Oral cholera vaccines (OCVs) are relatively new public health interventions, and limited data exist on the potential impact of OCV use on traditional cholera prevention and control measures—safe water, sanitation and hygiene (WaSH). To assess OCV acceptability and knowledge, attitudes, and practices (KAPs) regarding cholera and WaSH, we conducted cross-sectional surveys, 1 month before (baseline) and 3 and 12 months after (first and second follow-up) a preemptive OCV campaign in Maela, a long-standing refugee camp on the Thailand-Burma border. We randomly selected households for the surveys, and administered questionnaires to female heads of households. In total, 271 (77%), 187 (81%), and 199 (85%) households were included in the baseline, first and second follow-up surveys, respectively. Anticipated OCV acceptability was 97% at baseline, and 91% and 85% of household members were reported to have received 1 and 2 OCV doses at first follow-up. Compared with baseline, statistically significant differences (95% Wald confidence interval not overlapping zero) were noted at first and second follow-up among the proportions of respondents who correctly identified two or more means of cholera prevention (62% versus 78% and 80%), reported boiling or treating drinking water (19% versus 44% and 69%), and washing hands with soap (66% versus 77% and 85%); a significant difference was also observed in the proportion of households with soap available at handwashing areas (84% versus 90% and 95%), consistent with reported behaviors. No significant difference was noted in the proportion of households testing positive for *Escherichia coli* in stored household drinking water at second follow-up (39% versus 49% and 34%). Overall, we observed some positive, and no negative changes in cholera- and WaSH-related KAPs after an OCV campaign in Maela refugee camp. OCV campaigns may provide opportunities to reinforce beneficial WaSH-related KAPs for comprehensive cholera prevention and control.

## Author Summary

Safe water, sanitation, and hygiene (WaSH) are the primary measures for cholera prevention and control. Since 2010, oral cholera vaccines (OCVs) have been recommended as an additional tool for endemic and epidemic cholera prevention and control. Given the relatively new use of OCVs in public health programs, there is limited information on the impact of OCV use on traditional WaSH activities, i.e., can they serve as complementary tools, or will OCV use have a negative impact on WaSH-related behaviors? This study reports the findings of knowledge, attitudes and practices (KAP) surveys conducted before and after a preventive OCV campaign (2013) in a long-standing refugee camp in Thailand, where frequent cholera outbreaks had occurred in recent years. The surveys demonstrated high acceptability of the OCV campaign and several modest improvements in cholera and WaSH KAPs among the camp population. OCV campaigns may be used as opportunities to reinforce cholera and WaSH-related messaging towards strengthening comprehensive cholera prevention and control.

## Introduction

Cholera causes an estimated 2.9 million illnesses and 94,000 deaths annually, mostly in Asia and sub-Saharan Africa [1]. Cholera is caused by ingestion of food or water contaminated with feces containing toxigenic strains of *Vibrio cholerae* serogroups O1 and O139, and the illness is characterized by profuse watery diarrhea, vomiting, and dehydration. Case-fatality rates can exceed 70%, but can be as low as <1% if rehydration treatment is instituted rapidly and effectively [2].

Safe water, sanitation, and hygiene (WaSH), and effective cholera treatment are the mainstay of cholera prevention and control. In addition, two safe and effective oral cholera vaccines (OCV) are available globally; both are 2-dose, killed whole-cell vaccines. Shanchol (Shantha Biotechnics, India; now Sanofi) is a bivalent OCV (O1 and O139) that was first licensed in 2009 and prequalified by WHO in 2011 and is cheaper and easier to administer (i.e., not requiring water for buffer) than the previously licensed monovalent (O1) OCV. Moderate-to-high vaccine efficacy and field effectiveness has been demonstrated in multiple settings, including 67% efficacy over 5 years in an endemic area and 86% effectiveness during an acute outbreak [3, 4]. Since 2010, OCVs have been recommended by the World Health Organization (WHO) as an additional tool for control of endemic and epidemic cholera [5]. OCV campaigns have been conducted in multiple settings [6–14], including refugee camps in Uganda, South Sudan, and Tanzania [15–18].

In Maela, a long-standing refugee camp in Thailand, four cholera outbreaks occurred during 2005–2010, with an incidence of 0.7–10.7 cases per 1,000 refugees per year [19]. Multiple partners have conducted outbreak response activities which have included enhanced surveillance with active case finding, case management, and WASH interventions, including hygiene promotion, and distribution of soap, chlorine and covered water containers (C. Deglise, personal communication). After the 2010 outbreak, a decision was made to use OCV in Maela to further reduce the risk of cholera outbreaks in the camp [20]. During January–March 2013, a 2-dose OCV campaign with Shanchol was conducted, targeting all eligible 43,485 Maela residents (≥1 year of age and not pregnant) [19].

During 2010–2012, OCVs were a relatively new intervention, with limited data on the potential impact of vaccine use on WaSH measures, leading to concerns raised on the potential for OCV to detract from proven methods of cholera prevention and control [21]. In 2012, a WHO Technical Working Group on Creation of an OCV Stockpile recommended evaluation of OCV acceptability and impact on WaSH activities and created specific guidance on conducting knowledge, attitudes and practice (KAP) surveys during OCV campaigns [22, 23]. To assess the short- and long-term impact of an OCV intervention campaign and associated messaging on WaSH KAPs among the Maela camp population, we conducted household surveys 1 month before, and 3 and 12 months after the OCV campaign. We evaluated anticipated OCV acceptability before the campaign, campaign awareness and OCV uptake after the campaign, changes over time in reported knowledge, beliefs, and behaviors regarding cholera, WaSH and immunization. We also assessed changes in observed WaSH practices and stored household drinking water quality.

## Methods

### Study setting

#### Refugee camp

Maela camp was established in 1984, under management of the Royal Thai Government’s Ministry of the Interior, and provides shelter for approximately 46,000 refugees from Burma, predominately of Karen ethnicity [19]. Several essential services are provided by non-governmental organizations. At the time of the campaign and surveys, these organizations included The Border Consortium (TBC) (food and shelter rations), Première Urgence-Aide Médicale Internationale (PU-AMI) (clinical health services at two camp facilities, preventive care, public health, and outbreak response), and Solidarités International (SI) (WaSH services, including chlorinating and testing water sources, building latrines, and providing WaSH education and materials). Population density in the camp is high (11,500 persons per km^2^), and the existing infrastructure for improved water and sanitation is limited because of the intended temporary status of the camp [19]. One-third of camp residents use water sources, such as natural springs, private water networks, and water sellers, which are known to be less safe [19]. Mobility of the camp population is high [19].

#### OCV campaign and associated messaging

The 2-dose OCV campaign in Maela camp was implemented by PU-AMI with technical assistance by the U.S. Centers for Disease Control and Prevention (CDC) in three rounds during January–March 2013, with support from the Thailand Ministry of Public Health (MOPH) and the Bill and Melinda Gates Foundation [19]. Before the OCV campaign, barcoded vaccination cards with names were distributed to each camp resident. During the campaign, individual cards were scanned and manually date-stamped for each dose received; temporary cards were provided for anyone not bringing a vaccine card. A vaccination registry was created based on this documentation (18).

Immediately after the baseline KAP survey in December 2012, PU-AMI delivered information about the OCV campaign to camp leaders in meetings and to the community through posters, school presentations, loud-speaker announcements, and household visits, as described previously [19]. Documentation of campaign-associated messaging was reviewed and found to include reference to information about OCV (e.g., 2-dose requirement, limited vaccine efficacy and duration of immunity), handwashing, and cholera prevention practices (other than handwashing posters, documentation of specific cholera prevention message content was unavailable). Routine provision of WaSH services by SI (the water and sanitation NGO) continued per their mandate, and no additional WaSH interventions were conducted before, during, or immediately after the OCV campaign. Between 3 and 12 months following the campaign, SI conducted additional WaSH educational activities, including dramatic performances on cholera prevention, hand washing campaigns, and screenings of “The Story of Cholera” film by the Global Health Media Project (F. Cavalazzi, personal communication), but the extent of these activities could not be determined.

### Survey methodology

We conducted cross-sectional household-level surveys 1 month before the OCV campaign during November–December 2012 (baseline), 3 months after the campaign during May–June 2013 (first follow-up), and 12 months after the campaign in March 2014 (second follow-up). A household was defined as all persons living under one roof and sharing a “ration book” used for obtaining camp rations. For each survey, households were selected by simple random sampling from the most recent camp census, representing approximately 8,000 households [19]. All households living in the camp at least 1 month prior to the campaign were eligible for the survey. Households selected in the baseline or first follow-up survey remained eligible for selection in subsequent survey(s). Boarding houses for students were excluded from all surveys. Sample size was calculated using PASS 2008 (NCSS, Kaysville, UT) based on a Z-test (pooled variance) to detect a difference between two proportions as 350 households for the baseline and 200 households for both follow-up surveys, assuming a 50% baseline outcome and 15% change (α = 0.05, power = 89). Because non-response in the baseline survey was higher than expected (23% vs. 15%), the sample size of the follow-up surveys was revised to 233 households.

The survey was conducted by Maela refugees who were trained and working as PU-AMI community health workers (CHWs). Interviewers visited the household up to three times to attempt to enroll the female head of the household aged ≥18 years, or another adult if she was unavailable; non-responding households were not replaced. Interviews were conducted in Karen or Burmese language using a pre-tested, structured questionnaire including socio-demographic information, and KAPs regarding cholera, WaSH, immunization, and OCV acceptability; also included were observations about household characteristics, including water containers, handwashing stations, and latrines. OCV campaign vaccination status of all household members was first documented by recall of the household respondent and second, by review of available campaign vaccination cards.

### Ethics statement

This evaluation was determined to be a public health program evaluation activity according to the U.S. CDC’s human subjects’ procedures and approved by the Ethics Committee for Research in Human Subjects at the Thailand MOPH Department of Disease Control. Written informed consent was collected from all participants by signature or thumb print. Only adults aged ≥18 years were intended to be interviewed, but in some cases, interviewers mistakenly enrolled female heads of household aged <18 years. In Burma, the country of origin for survey interviewers and participants, the age of legal adulthood is 16 years, rather than 18 years, and most of the under-age respondents were ≥16 years old. While data on marital status was not collected during the survey, it is likely that these women, who self-identified themselves as female heads of households, were married women. In several countries including the United States, marriage is a special circumstance for minors to be considered as 'emancipated minors' (legally considered adults). Hence, no attempt was made to obtain consent from the parents of these women.

### Laboratory methods

As an objective measure of drinking water quality, stored household drinking water was collected and tested for residual chlorine and fecal contamination on the day of the survey visit. Interviewers tested a 5 ml water sample for residual chlorine using N, N diethyl-p-phenylenediamine sulfate (LaMotte, Chestertown, MD) and collected a 100 ml water sample in a Whirl-Pak bag with sodium thiosulfate (Nasco, Fort Atkinson, WI), using sterile technique. The second sample was transported on ice (2‒8°C) for off-site microbial testing using Colisure media (IDEXX Laboratories, Westbrook, ME). After incubating for 24–48 hours at 35°C, samples were recorded as positive or negative for *Escherichia coli*, per the manufacturer’s protocol.

### Data analysis

Data were analyzed using SAS version 9.3 (SAS Institute, Cary, NC). We performed a descriptive analysis of the baseline survey, including socio-demographic characteristics and cholera, WaSH, and immunization KAPs. The first and second follow-up surveys were each compared with baseline. For binary outcomes, we calculated absolute differences in proportions with 95% Wald asymptotic confidence intervals (CIs); differences were judged to be significant if their CIs did not overlap zero. For continuous outcomes, we used Wilcoxon two-sample tests. OCV coverage estimates and 95% Wilson CI were calculated using Taylor series linearization method to account for intra-household correlation. Definitions for constructed variables such as “knowing two or more means of cholera transmission” are given as footnotes in the tables.

## Results

### Characteristics of the survey samples

A total of 271 (77%), 187 (81%) and 199 (85%) households were enrolled in the baseline, first follow-up and second follow-up surveys, respectively. Reasons for survey non-response are listed in Table 1. At baseline, median duration of camp residency was 8 years (range: 1–31), and median household size was 5 persons (range: 1–15). Among survey respondents, 206 (77%) were female with a median age of 39 years (range: 15–77 years); 213 (79%) were of Karen ethnicity, and 107 (40%) had never attended school. Overall, 44% of households had electricity, and most used squat or pour-flush toilets (74% and 16%, respectively) (Table 2).

**Table 1:** Reasons for household non-response in the surveys conducted 1 month before (baseline), and 3 and 12 months after (first and second follow-up) an oral cholera vaccination campaign, Maela Camp, 2013.

Reason for non-response	Baseline		1^st^ follow-up		2^nd^ follow-up	
	No.	%	No.	%	No.	%
Temporarily lived/worked outside camp	32	40	10	22	16	47
No longer lived in camp	28	35	12	26	14	41
Not home	15	19	11	24	1	3
Could not be located	2	3	11	24	2	6
Refused	-	-	1	2	1	3
Ineligible (arrived <1 month before campaign)	-	-	1	2	-	-
Reason not recorded	2	3	-	-	-	-
**Total**	79	100	46	100	34	100

**Table 2:** Socio-demographic characteristics of respondent households in the surveys conducted 1 month before (baseline), and 3 and 12 months after (first and second follow-up) an oral cholera vaccination campaign, Maela Camp, 2013.

Characteristic	Baseline		1^st^ follow-up		2^nd^ follow-up	
	(n = 271)^a^		(n = 187)^a^		(n = 199)^a^	
	No.	%	No.	%	No.	%
Households with child aged <5 years	141	52	101	54	108	54
Households with child aged 5–14 years	190	70	135	72	137	69
Female	206	77	142	76	153	77
Ethnicity^b^						
Karen	213	79	156	84	165	83
Burmese	11	4	0	0	1	1
Muslim^c^	36	13	24	13	28	14
Other	11	4	6	3	5	3
Education^d^						
No school	107	40	67	36	78	39
Some primary school	90	33	44	24	61	31
Some middle school	29	11	28	15	28	14
Some high school or higher	44	16	45	24	30	15
Electricity	118	44	79	42	101	51
Own TV	104	39	64	35	92	46
Own mobile phone	218	80	164	88	163	82
Share a toilet with other households	16	6	17	9	20	10
Toilet^e^						
Pour flush toilet	43	16	38	20	24	12
Squat toilet	201	74	120	64	157	79
Pit latrine	22	8	24	13	15	8
Other	4	1	5	3	3	2
	**Median (range)**		**Median (range)**		**Median (range)**	
Median duration of camp residency (years)	8 (1–31)		8 (1–33)		9 (1–29)	
Median household size	5 (1–15)		5 (1–26)		6 (1–23)	
Median age (years)	39 (15–77)		38 (17–88)		38 (16–76)	

^a^ Missing data resulted in small fluctuations in denominators for some responses

^b^ For statistical analysis, ethnicity was computed as a binary outcome (Karen vs. non-Karen)

^c^ In the camp, "Muslim" is a widely identified ethnicity, in addition to being a religion

^d^ For statistical analysis, education was computed as a binary outcome (some school vs. no school)

^e^ For statistical analysis, toilet was computed as a binary outcome (pour flush and squat toilet vs. pit latrine and other)

No differences were observed between the baseline and first or second follow-up surveys in household demographic characteristics or ownership of most durable consumer goods (Table 2). The proportion of households owning a mobile phone increased 8% (95% CI: 1%–14%) between the baseline and first follow-up survey; because mobile phone ownership was not deemed to be a primary indicator of socio-economic status for a household, an adjusted analysis was not performed.

### Cholera and WaSH knowledge

At baseline, 218 (81%) of 270 respondents had heard about cholera; of these, 114 (52%) mentioned watery diarrhea as a symptom of cholera. Overall, 133 (61%) of 218 respondents knew two or more vehicles of cholera transmission, and 136 (62%) knew two or more means of cholera prevention. Most respondents (97%) reported they would go to the camp clinic for cholera treatment; no respondents mentioned using oral rehydration solution (ORS). A total of 182 (83%) of 218 respondents reported having heard about cholera prevention and treatment from other people or the media; of those, most (77%) received the information from health workers visiting the home, and most (91%) received materials for protecting their household against cholera (Table 3).

**Table 3:** Knowledge and practices about safe water, sanitation and hygiene in surveys conducted 1 month before (baseline), and 3 and 12 months after (first and second follow-up) an oral cholera vaccination campaign, Maela Camp, 2013.

Knowledge	Baseline		1^st^ follow-up		2^nd^ follow-up	
	(n = 271)^a^		(n = 187)^a^		(n = 199)^a^	
	No.	%	No.	%	No.	%
Heard of cholera	218	81	152	81	181	91
Knew that watery diarrhea was a symptom of cholera	114	52	68	45	95	52
Knew ≥2 vehicles of cholera transmission^b^	133	61	90	59	148	82
Knew ≥2 means of cholera prevention^c^	136	62	118	78	144	80
Knew about boiling or treating drinking water for cholera prevention	115	53	100	66	96	53
Knew about washing hands with soap for cholera prevention	107	49	66	43	97	54
Knew about cooking food thoroughly for cholera prevention	101	46	78	51	103	57
Reported would go to clinic for cholera treatment	211	97	151	99	181	100
Reported would use oral rehydration for cholera treatment	0	0	10	7	10	6
Heard about preventing and treating cholera from people or media	182	83	124	82	166	92
Received soap to prevent cholera	150	83	94	76	135	81
Received chlorine solution to prevent cholera	62	34	15	12	28	17
Received water container to prevent cholera	40	22	8	6	23	14
Received printed educational materials to prevent cholera	18	10	3	2	49	30
**Practice**						
Reported camp water tap as primary drinking water source	180	67	144	77	138	70
Reported boils or treats drinking water^d^	51	19	83	44	137	69
Reported boiling or treating drinking water within last 24 hours^d^	32	12	54	29	122	61
Reported washing hands ≥3 types of occasions^e^	133	49	93	50	134	67
Reported washing hands before eating and after using the toilet	124	46	99	53	123	62
Reported using soap to wash hands	178	66	143	77	170	85
Observed soap at hand-washing station	227	84	168	90	188	95
Observed covered drinking water container with spigot	195	73	118	63	130	65
Observed residual chlorine in household drinking water sample	22	8	8	4	3	2
Observed *E*. *coli* in household drinking water sample	106	39	91	49	68	34

^a^ Missing data resulted in small fluctuations in denominators for some responses

^b^ Drinking bad water, eating bad food, eating foods or drinks prepared outside the home, not washing fruits and vegetables, not cooking food thoroughly, flies/insects, poor hygiene/hand-washing, and eating raw fish

^c^ Wash hands with soap and water, cook food thoroughly, drink water from public tap, boil/filter drinking water, treat water with chlorine, wash fruits/vegetables, clean cooking utensils/vessels, dispose of human waste properly, and cover food to keep away flies

^d^ Limited comparibility between baseline and 3-month follow-up, or baseline and 1 year follow-up due to changes in translation made after the baseline survey

^e^ After using toilet, after washing/cleaning tables, before eating, after eating, after cleaning baby diapers/stools, before cooking

**Fig 1:**
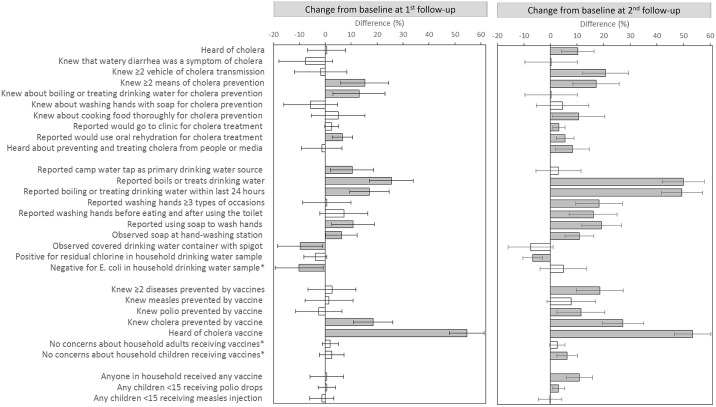
Differences in knowledge, attitudes and practices (KAPs) for surveys conducted 1 month before (baseline) versus 3 and 12 months after (first and second follow-up) an oral cholera vaccination campaign in Maela Camp, 2013. Absolute differences in proportions for KAP responses in the first and second follow-up surveys compared to baseline were calculated; error bars depict 95% Wald asymptotic confidence interval (CI). Statistically significant differences (CI not overlapping zero) are shown in grey; non-significant differences are shown in white. Outcomes marked with * have directionality of difference switched from Tables 3 and 4, so that improvements are depicted in positive direction, and negative changes are depicted in negative direction.

Compared with baseline, the proportion of respondents in the first follow-up survey who knew two or more means of cholera prevention increased 15% (95% CI: 6%–24%), and those who knew about boiling or treating drinking water for cholera prevention increased 13% (95% CI: 3%–23%). The proportion who reported they would use ORS for cholera treatment increased 7% (95% CI: 3%–11%) (Table 3 and Fig 1). Though the proportion of respondents who reported hearing about cholera prevention and treatment from other people or the media was unchanged, reported receipt of materials to prevent cholera decreased, including receipt of chlorine solution (22% [95% CI: 13%–33%]), water containers (16% [95% CI: 8%–23%]), and printed education materials (8% [95% CI: 2%–13%]); reported receipt of soap was unchanged (Table 3).

In the second follow-up survey, changes in cholera and WaSH knowledge that were sustained from the first follow-up survey included a 17% (95% CI: 8%–26%) increase in the proportion of respondents who knew two or more means of cholera prevention; a 6% (95% CI: 2%–9%) increase in those reporting they would use ORS for cholera treatment; and a 17% (95% CI: 8%–26%) and 8% (95% CI: 0%–16%) decrease in reported receipt of chlorine solution and water containers compared to baseline, respectively. Changes observed in the second follow-up survey, but not in the first follow-up survey, included that 10% (95% CI: 4%–16%) more respondents reported having heard about cholera; 21% (95% CI: 12%–29%) more mentioned two or more vehicles of cholera transmission; 3% (95% CI: 1%–6%) more reported they would go to the clinic for cholera treatment; and 8% (95% CI: 2%–15%) more reported having heard about cholera prevention and treatment from other people or the media, with 20% (95% CI: 11%–28%) more reporting receipt of printed materials, compared with baseline (Table 3 and Fig 1).

### WaSH practices

At baseline, 180 (67%) of 270 respondents reported the camp public tap as their primary household drinking water source. Overall, 51 (19%) households reported boiling or treating drinking water to make it safer, and 32 (12%) reported doing so in the last 24 hours. For households that reported not boiling or treating their water, 89% reported that they believed that their “water source is safe and does not need treatment.” A total of 133 (49%) respondents reported washing hands on at least three types of occasions, and 178 (66%) reported using soap to wash hands; soap was observed at the place for handwashing in 227 (84%) households. Safe water containers, defined as covered water containers with spigots, were observed in 195 (73%) of 268 households. Among the 271 households surveyed, residual chlorine was detected in 22 (8%) households’ stored drinking water and *E*. *coli* contamination was detected in 106 (39%) (Table 3).

Compared with baseline, 10% (95% CI: 2%–19%) more respondents in the first follow-up survey reported using the camp public tap as their primary drinking water source. The proportion of households that reported boiling or treating drinking water increased 25% (95% CI: 17%–34%), and those that reported doing so in the last 24 hours increased 17% (95% CI: 9%–25%); however, a difference in translation for these questions between the baseline and follow-up surveys limits comparability. Reported use of soap to wash hands increased 11% (95% CI: 2%–19%), and observed soap at handwashing stations increased 6% (95% CI: 0%–12%). At first follow-up compared with baseline, safe water containers were observed in 10% (95% CI: 1%–18%) fewer households, and 10% (95% CI: 1%–19%) more households had *E*. *coli* contamination of stored drinking water (Table 3 and Fig 1). *E*. *coli* contamination of stored household drinking water was associated with a lack of safe water containers in the first follow-up survey (p = 0.006).

In the second follow-up survey, changes in WaSH knowledge and practices that were sustained from the first follow-up survey included a 50% (95% CI: 42%–58%) increase in the proportion who reported boiling or treating their drinking water and a 50% (95% CI: 41%–57%) increase in those who reported doing so in the last 24 hours (between the first and second follow-up surveys, where translation of these questions was comparable, increases of 24% [95% CI: 15–34%] and 32% [95% CI: 23–42%] were observed, respectively). Other sustained changes in the second follow-up survey, compared with baseline, were a 19% (95% CI: 12%–27%) increase in reported use of soap to wash hands and an 11% (95% CI: 6%–16%) increase in soap observed at handwashing stations. Changes observed in the second follow-up survey, but not in the first follow-up survey, included 18% (95% CI: 9%–27%) more respondents who reported washing hands on at least three types of occasions and 7% (95% CI: 3%–10%) fewer households with residual chlorine detected in stored drinking water samples, compared with baseline (Table 3 and Fig 1).

### Vaccine knowledge, attitudes, and practices

At baseline, 135 (50%) of 271 respondents could name two or more diseases prevented by vaccines, and 107 (39%) respondents claimed to have heard of “cholera vaccine.” Overall, 11 (4%) respondents reported having any concerns about adults in the household receiving vaccines, and 17 (7%) reported concerns about children in the household receiving vaccines. In total, 231 (86%) of 271 respondents reported a household member receiving any vaccine; 220 (97%) and 217 (95%) of 228 respondents reported a child in the household receiving polio drops and a measles injection, respectively (Table 4).

**Table 4:** Knowledge, attitudes and practices about vaccination in surveys conducted 1 month before (baseline), and 3 and 12 months after (first and second follow-up) an oral cholera vaccination campaign, Maela Camp, 2013.

Knowledge and attitude	Baseline		1^st^ follow-up		2^nd^ follow-up	
	(n = 271)^a^		(n = 187)^a^		(n = 199)^a^	
	No.	%	No.	%	No.	%
Knew ≥2 diseases prevented by vaccines^b^	135	50	98	52	136	68
Knew measles prevented by vaccine	115	42	82	44	100	50
Knew polio prevented by vaccine	101	37	65	35	97	49
Knew cholera prevented by vaccine	31	11	56	30	77	39
Heard of cholera vaccine	107	39	176	94	185	93
Concerns about household adults receiving vaccines	11	4	4	2	3	2
Concerns about household children receiving vaccines^c^	17	7	8	5	2	1
**Practice**						
Reported household member received any vaccine	231	86	161	86	192	96
Reported child aged <15 years received polio drops^c^	220	97	158	98	161	100
Reported child aged <15 years received measles injection^c^	217	95	152	94	153	95

^a^ Missing data resulted in small fluctuations in denominators for some responses

^b^ Cholera, diarrhea, tuberculosis, hepatitis, diphtheria, pneumonia, tetanus, meningitis, whooping cough (pertussis), measles, polio, chickenpox, and typhoid

^c^ Number of households without children was 43 in baseline, 38 in 1st follow-up, and 25 in 2nd follow-up

Compared with baseline, the proportion of respondents in the first follow-up survey mentioning cholera as a disease they knew to be prevented by vaccine increased 19% (95% CI: 11%–26%), and those reporting they had heard of “cholera vaccine” increased 55% (95% CI: 48%–61%). In the second follow-up survey, changes in vaccine KAPs that were sustained from the first follow-up survey relative to baseline included a 27% (95% CI: 19%–35%) increase in the proportion of respondents who named cholera as a disease prevented by vaccine, and a 53% (95% CI: 47%–60%) increase in those who had heard of “cholera vaccine.” Changes observed in the second follow-up survey, but not in the first follow-up survey, included 19% (95% CI: 10%–27%) more respondents mentioning two or more diseases prevented by vaccines and 6% (95% CI: 2%–10%) fewer respondents reporting concerns about children in the household receiving vaccines, compared to baseline.

#### OCV acceptability and uptake

At baseline, 262 (97%) of 271 respondents reported willingness to receive OCV, and 221 (97%) of 228 of respondents with children reported willingness to let their child receive OCV. At first follow-up, 184 (99%) of 187 respondents reporting awareness of the OCV campaign. The most commonly reported source of information about the campaign was health workers visiting the home (45%). When respondents were questioned about the duration and level of protection of OCV, 86 (49%) expected up to 2 years, and 23 (13%) expected 3–5 years of protection from cholera; 101 (54%) expected the vaccine would be “somewhat protective, with other protective measures needed” (Table 5).

**Table 5:** Oral cholera vaccination (OCV) acceptability 1 month before the campaign (baseline) and campaign awareness and OCV uptake 3 months after the campaign (first follow-up), Maela Camp, 2013.

Baseline (n = 271)	No.	%
Willing to receive cholera vaccine	262	97
Willing to let child receive cholera vaccine^a^	221	97
**1st follow-up (n = 187)**^b^		
Heard of cholera vaccine campaign	184	99
Health worker visiting home	85	45
Section leader	68	36
Staff at the clinic	48	26
Megaphone/loudspeaker	23	12
Neighbor or friend	22	12
Poster	21	11
A meeting in the camp	18	10
Other	6	3
Expected duration of protection of cholera vaccine		
Up to 2 years	86	49
From 3–5 years	23	13
From 6–10 years	4	2
Lifetime	24	14
Don't know	39	22
Expected level of protection of vaccine		
Completely protective, no other measures needed	80	43
Somewhat protective, some other measures needed	101	54
Not protective, other measures needed	1	1
No measures can prevent cholera	-	-
Don't know	4	2
Reported household member received vaccine	186	99
Reported household member didn't receive vaccine	70	38
Not old enough for vaccine	30	16
Absence	26	14
Pregnant during campaign	14	7
Busy/no time	10	5
Sick during campaign	7	4
Bad taste of vaccine	1	1
Other	7	4
Reported household member received only one dose	35	19
Absence	12	6
Busy/no time	6	3
Sick during campaign	6	3
Adverse event after 1st dose	4	2
Forgot to go	3	2
Bad taste of vaccine	2	1
Other	6	3
Reported household member spit out part of vaccine	2	1
Reported household member had adverse event	17	9
Dizziness/loss of balance	8	4
Headache	5	3
Nausea	4	2
Fever	3	2
Abdominal pain	1	1
Weakness/fatigue	2	1

^a^ No. of households without children = 43

^b^ Missing data resulted in small fluctuations in denominators for some responses

Overall, 186 (99%) of respondents reported at least one household member receiving OCV during the campaign; 70 (38%) reported non-vaccination of at least one household member; and 35 (19%) reported at least one household member receiving only 1 dose. Absence from the camp was the most commonly reported reason both for non-vaccination (14%) and receipt of only 1 OCV dose (6%), besides non-eligibility. Few (1%) respondents reported a household member spitting out part of the vaccine. Overall, 17 (9%) of 187 respondents reported adverse events following campaign vaccination in a household member (Table 5).

Overall, OCV coverage by respondents’ recall was 91% (95% CI: 88%‒93%) for the first dose and 85% (95% CI: 81%‒89%) for the second dose, among the 1,102 individuals aged ≥1 year in households surveyed at first follow-up. Vaccination cards were available for 443 (40%) individuals. OCV coverage by card, among individuals with available documentation, was 93% (95% CI: 89%‒96%) for the first dose and 84% (95% CI: 78%‒89%) for the second dose (Table 6). Assuming that all 203 individuals in the 46 non-responding households were unvaccinated, OCV coverage in the camp overall could have been as low as 77% (95% CI: 72%‒82%) and 72% (95% CI: 67%‒77%) for the first and second dose, respectively.

**Table 6:** Oral cholera vaccine (OCV) coverage by survey of household respondents’ recall and individual vaccination cards 3 months after an OCV campaign (first follow-up), Maela Camp, 2013.

Source^a^	Age group (years)	Total	First dose coverage				Second dose coverage			
Recall		No.	No.	%	LCL	UCL	No.	%	LCL	UCL
	1–4	112	106	95	88	98	95	85	75	91
	5–14	307	297	97	91	99	287	93	88	97
	≥15	683	599	88	84	91	557	82	77	85
	Total	1102	1002	91	88	93	939	85	81	89
**Card**										
	1–4	55	53	96	87	99	47	85	72	93
	5–14	126	122	97	91	99	114	90	83	95
	≥15	262	239	91	86	95	212	81	74	86
	Total	443	414	93	89	96	373	84	78	89

Abbreviations: LCL = lower confidence limit; UCL = upper confidence limit

^a^ Household respondents' recall was collected for all household members first, followed by reviewing individual vaccination cards, ("card" responses are the subset of "recall" with available documentation)

## Discussion

To our knowledge, this was the first evaluation of the short and long-term impact of an OCV campaign and associated messaging on KAPs regarding cholera, WaSH, and OCV through inclusion of surveys at three time points (1 month before, and 3 and 12 months after the OCV campaign). It was also the first KAP evaluation of a Shanchol OCV campaign in a long-standing refugee camp. Despite repeated outbreaks and outbreak response efforts in Maela refugee camp during 2005–2010 [19], baseline cholera knowledge and safe water practices were generally low (45–70%). At first follow-up, our results showed modest improvements (6–15% increase) in knowledge of cholera prevention and treatment, as well as reported use and observation of soap for handwashing in the household, in the absence of a change in the reported receipt of soap for cholera prevention. These improvements were sustained at second follow-up, with additional positive and no negative long-term changes noted.

Several other cross-sectional evaluations conducted at single time points in endemic and epidemic cholera settings have similarly documented varying WaSH KAPs [24–26]. The improvements we observed at first follow-up survey after the OCV campaign were similar to that of a KAP evaluation in Haiti conducted before and 3 months after an OCV campaign in 2012, the only other pre- and post-campaign KAP evaluation [27]. However, the improvements in cholera knowledge and WaSH behaviors noted in Haiti were more widespread and pronounced, which may be explained by a more robust cholera and WaSH educational component included in the Haiti OCV campaign than in the Maela campaign [27]. A KAP study in the Solomon Islands also demonstrated high cholera prevention knowledge and behaviors after WaSH messaging delivered during a targeted OCV campaign [28].

In Maela, the modest KAP improvements at first follow-up generally correlated with the focus areas for campaign messaging—cholera prevention and handwashing. The majority of respondents received the proper messaging concerning the limited duration and protection of OCV, another focus of campaign messaging. More improvements in WaSH knowledge and practices were observed at second follow-up than first follow-up compared with baseline; likewise, reports of hearing about and receiving printed educational materials for cholera prevention increased. The contribution of the OCV campaign to improvements in WaSH KAPs occurring between the first and second follow-up surveys could not be evaluated relative to the WaSH educational activities documented to have been conducted by SI during this timeframe. It is possible that in Maela and similar settings with ongoing WaSH activities (e.g., home visits, dramas), high-profile OCV campaigns may provide increased visibility to cholera-related issues that may contribute to people remembering more after the campaign, thereby reinforcing repeated WaSH messaging.

The high anticipated acceptability (97%) and OCV campaign coverage (first dose: 91%; second dose: 85%) observed were similar to evaluations from other OCV campaigns [12, 14, 16, 18, 24, 29–33], but higher than previously published coverage estimates from the Maela OCV campaign based on a vaccination registry (first dose: 83%; second dose: 61%) [19]. Although assessing vaccination history from recall may introduce bias [34], we observed similarly high coverage (first dose: 93%; second dose: 84%) among those retaining vaccination cards distributed before the campaign. Using the most recent census for our survey sampling frame, we had a high proportion of non-responding households (19%), who may have been more likely to miss campaign vaccination due to absence. Campaign coverage estimates from the vaccination registry were more similar to survey estimates calculated under a scenario where all members of non-responding households were considered as unvaccinated (first dose: 77%; second dose: 72%). Results highlight the challenge of working with mobile populations and suggest OCV campaign coverage among the population continuously residing in the camp may be higher than previously estimated.

We observed several temporary changes at the first follow-up survey not sustained at the second follow-up, including decreased observation of safe water containers, increased reported use of the camp tap as the primary drinking water source, and increased *E*. *coli* contamination of stored household drinking water. Fluctuations in availability of WaSH materials, such as safe water containers, can occur based on variations in the timing and frequency of their distribution within the camp. While increased fecal contamination of stored drinking water at first follow-up was correlated with decreased use of safe water containers in households, the temporary increase could alternatively reflect the shift to the rainy season during May–June that marks increased opportunities for water contamination and increased use of unsafe water sources in the camp (F. Cavalazzi, personal communication). *E*. *coli* contamination was moderately high and similar between baseline and second follow-up surveys (both occurring in the dry season), and the amount of residual chlorine detected in stored drinking water was low in all three surveys; these results suggest no long-term change in practices which has also been seen in other studies [35–38]. While conducting a KAP survey soon after the OCV campaign was important for assessing the temporality of changes, environmental factors and WaSH behaviors may be impacted by seasonal trends, making assessment of OCV campaign-associated changes difficult [27].

Our study had some limitations. WaSH KAPs are influenced by multiple factors, and a direct attribution of observed changes to the OCV campaign is not possible. Social desirability bias may have led to reporting favorable practices to interviewers who work as CHWs in the camp. Non-response bias, due to frequent absence and relocation of camp residents, may have affected outcomes in either direction. Accurate translation of the questionnaire into Burmese and Karen was made difficult by the existence of multiple dialects and the low education level among residents in the camp. Despite intensive translation efforts, we discovered one issue after the baseline survey where translation of the lead-in question “do you do anything to your drinking water to make it safer?” implied adding something to the water, which may have resulted in underestimation of water boiling at baseline. The words for “cholera” and “diarrhea” are similar in these languages, which may have led to some challenges with interpretation; however, analysis of responses to equivalent questions for diarrhea and cholera in the baseline survey suggested that respondents might have found the questions to be redundant rather than challenging to interpret.

This study demonstrates modest improvements in some reported WaSH-related KAPs and observed handwashing practices after an OCV campaign in a long-standing refugee camp in Thailand, both in the short- and long-term (3 and 12 months post-campaign). WaSH remains the mainstay of cholera prevention and control, but providing OCVs in conjunction with WaSH interventions may be particularly useful in settings with limited safe water and sanitation infrastructure, such as refugee camps. OCV use has expanded post-licensure, due in part to WHO initiating an OCV stockpile for epidemic response in 2012, and Gavi, the Vaccine Alliance providing a financial contribution in 2013 to enable use in low-income countries [39]. Since OCV campaigns have the potential to be high-profile activities with high acceptability and uptake, we recommend planned integration of strong WaSH messaging towards achieving comprehensive cholera prevention and control in high-risk communities globally. Further pre- and post-evaluations of the impact of OCV campaigns on WaSH KAPs are needed from other settings [22]. We recommend including household observations and other objective measures to distinguish changes in reported KAPs from actual behavior changes.

## Supporting Information

S1 Checklist STROBE Checklist. (DOC)
