# Correction: Use of Oral Cholera Vaccine and Knowledge, Attitudes, and Practices Regarding Safe Water, Sanitation and Hygiene in a Long-Standing Refugee Camp, Thailand, 2012-2014

**DOI:** 10.1371/journal.pntd.0005810

**Published:** 2017-07-28

**Authors:** Heather M. Scobie, Christina R. Phares, Kathleen A. Wannemuehler, Edith Nyangoma, Eboni M. Taylor, Anna Fulton, Nuttapong Wongjindanon, Naw Rody Aung, Philippe Travers, Kashmira Date

The ninth author's name is spelled incorrectly. The correct name is: Philippe Travers.
